# A pilot investigation to optimise methods for a future satiety preload study

**DOI:** 10.1186/s40814-017-0208-x

**Published:** 2017-11-17

**Authors:** Mark R. Hobden, Laetitia Guérin-Deremaux, Daniel M. Commane, Ian Rowland, Glenn R. Gibson, Orla B. Kennedy

**Affiliations:** 1 Department of Food and Nutritional Sciences, School of Chemistry, Food and Pharmacy, The University of Reading, Reading, RG6 6AP UK; 2 Department of Nutrition and Health, Roquette, Lestrem, France

**Keywords:** Appetite, Preload, Satiety, Study design, Optimisation

## Abstract

**Background:**

Preload studies are used to investigate the satiating effects of foods and food ingredients. However, the design of preload studies is complex, with many methodological considerations influencing appetite responses. The aim of this pilot investigation was to determine acceptability, and optimise methods, for a future satiety preload study. Specifically, we investigated the effects of altering (i) energy intake at a standardised breakfast (gender-specific or non-gender specific), and (ii) the duration between mid-morning preload and ad libitum lunch meal, on morning appetite scores and energy intake at lunch.

**Methods:**

Participants attended a single study visit. Female participants consumed a 214-kcal breakfast (*n* = 10) or 266-kcal breakfast (*n* = 10), equivalent to 10% of recommended daily energy intakes for females and males, respectively. Male participants (*n* = 20) consumed a 266-kcal breakfast. All participants received a 250-ml orange juice preload 2 h after breakfast. The impact of different study timings was evaluated in male participants, with 10 males following one protocol (protocol 1) and 10 males following another (protocol 2). The duration between preload and ad libitum lunch meal was 2 h (protocol 1) or 2.5 h (protocol 2), with the ad libitum lunch meal provided at 12.00 or 13.00, respectively. All female participants followed protocol 2. Visual analogue scale (VAS) questionnaires were used to assess appetite responses and food/drink palatability.

**Results:**

Correlation between male and female appetite scores was higher with the provision of a gender-specific breakfast, compared to non-gender-specific breakfast (Pearson correlation of 0.747 and 0.479, respectively). No differences in subjective appetite or ad libitum energy intake were found between protocols 1 and 2. VAS mean ratings of liking, enjoyment, and palatability were all > 66 out of 100 mm for breakfast, preload, and lunch meals.

**Conclusions:**

The findings of this pilot study confirm the acceptability of this methodology for future satiety preload studies. Appetite scores increased from preload to ad libitum lunch meal; however, no specific differences were found between protocols. The results highlight the importance of considering energy intake prior to preload provision, with a gender-specific breakfast improving the correlation between male and female appetite score responses to a morning preload.

## Background

There is appreciation for the importance of intervention development, and pilot/feasibility, studies in scientific research [1]. Until recently, much of this preliminary work, including decision-making processes, methods optimisation and formal testing prior to a full-scale study, has gone unreported [2]. However, data generated from such studies is of interest to the scientific community, most notably for those individuals undertaking similar research. Pilot studies provide data to promote scientific rigour and ensure that future study protocols are optimised to best investigate their aims and objectives [3].

Illnesses associated with lifestyle factors, such as unhealthy eating habits and behaviours, are estimated to be responsible for 40% of all deaths in the UK and cost the National Health Service in excess of £11 billion each year [4]. Accordingly, the effects of food and food ingredients on satiety and weight management are a key focus in nutrition research [5]. Preload study designs, in which the food or ingredient of interest is consumed a fixed duration before the provision of an ad libitum meal, are often used [6]. Satiety, defined as ‘the feeling of fullness that persists after eating, potentially suppressing further energy intake until hunger returns’, can be measured using subjective ratings and ad libitum meals [5]. When designing preload studies, researchers must decide on the most suitable fixed duration between preload and the ad libitum meal. This decision is influenced by previous knowledge of the satiating effects of the test product and/or an understanding of the theorised physiological mechanisms of action [7]. Moreover, another important consideration is what time the ad libitum meal is provided in relation to habitual intake and circadian body clock, with subjective appetite ratings and plasma ghrelin concentrations known to correlate with habitual meal timings and rise in anticipation of eating [8].

Researchers must also consider what foods and/or drinks are to be consumed by participants before the preload. Not all study designs involve food/drink consumption before preload provision, however, in certain study designs a standardised meal is given before preload. For example, if the preload is consumed mid-morning then a standardised breakfast is often provided. Many previous studies have used the same breakfast for all participants, irrespective of gender or individual differences [9, 10]. However, recommended dietary energy intakes are higher for males (adult men, 2605 kcal/day) than females (adult women, 2079 kcal/day) [11]. Furthermore, post-prandial appetite responses to a standardised meal have been shown to differ between men and women [12]. Accordingly, it can be postulated that altering the amount of energy provided at a fixed meal prior to preload provision, to reflect recommended energy intakes by gender, may improve the correlation between men and women for appetite and energy intake responses. Indeed, this might be especially important for breakfast, with evidence that breakfast meal choice and specifically, differences in energy/macronutrient content and physical size, may impact on subsequent appetite and ad libitum energy intake [13–15]. To our knowledge, no studies to date have investigated the comparative effects of providing a gender-specific or non-gender-specific energy intake at breakfast on appetite responses and ad libitum energy intake.

## Methods

The current pilot study was conducted to optimise methods for a future human intervention study investigating the effects of a non-digestible carbohydrate on satiety. The study was given ethical approval from the University of Reading Ethics Committee (11/08) and followed principles outlined in the Declaration of Helsinki (2008).

The specific aims of this pilot study were to investigate the appetite regulatory effects of:

- altering energy provision at a standardised breakfast to reflect gender-specific differences in recommended daily energy intakes
- adjusting the duration between preload and ad libitum lunch meal (2 or 2.5 h), and the time of ad libitum lunch meal provision (12.00 or 13.00)

The following were evaluated to ascertain the acceptability of the study design: data collection forms and questionnaires, suitability of the clinical unit environment, study visit logistics, palatability and enjoyment of the study meals.

### Study design and recruitment

**Fig. 1:**
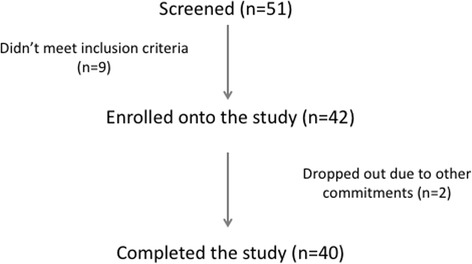
Participant flow diagram

The pilot study was a randomised, acute study, involving one study visit. Participants were recruited from Berkshire, UK through advertising, and an existing participant database, between November 2011 and April 2012. Participants provided informed written consent before enrolment on the study. Inclusion criteria were body mass index (BMI) 21–30 kg/m^2^; free of disease and currently not taking any medication (excluding contraceptives), no antibiotic usage for > 6 months prior to beginning the study, and resting blood pressure (< 160/90 mmHg). Participants were non-smokers and bodyweight stable. Female participants self-reported as non-pregnant. Participants had no self-reported history of alcohol or drug misuse. Participants also completed a three-factor eating questionnaire (TFEQ) and were included if they had a cognitive restrain < 13 [16, 17]. This cut off was chosen to exclude individuals with high restraint scores, as this may have affected food intake at the ad libitum meal. As shown in Fig. 1, 51 participants were screened for suitability, 42 participants were enrolled into the study and 40 participants completed the study. Two participants withdrew prior to the study visit due to other commitments.

### Study groups

**Fig. 2:**
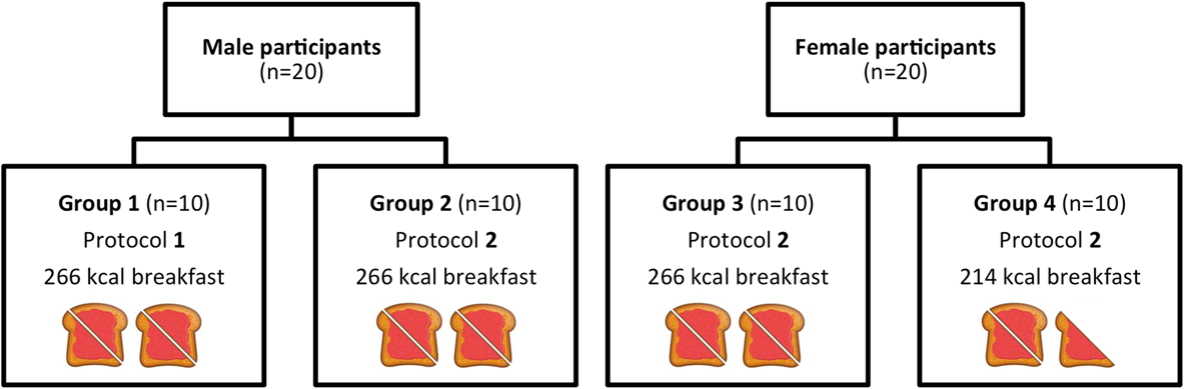
Participant study group allocation. Male participants allocated to groups 1 or 2 and female participants allocated to groups 3 or 4. *n* refers to the number of participants in each group. Group 1: male participants, 266 kcal breakfast, protocol 1. Group 2: male participants, 266 kcal breakfast, protocol 2. Group 3: female participants, 266 kcal breakfast, protocol 2. Group 4: female participants, 214 kcal breakfast, protocol 2

Once enrolled onto the study, male participants were randomised to study group 1 or 2 and female participants were randomised to study group 3 or 4 (Fig. 2). The randomisation scheme was generated online (randomization.com) using the block method and no specific stratification.

### Study visit

**Fig. 3:**
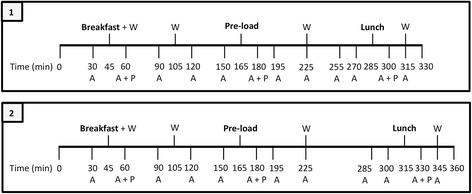
Schematic representation of the two protocols used in the study. Group 1 followed protocol 1 and groups 2, 3 and 4 followed protocol 2. Water was provided in 200 ml servings. A = appetite VAS questionnaire; P = study food/drink palatability VAS questionnaire; W = water

Participants were asked to consume a standardised evening meal before 8 pm the day before the study visit. This was a macaroni cheese ready meal (755 kcal). Participants were also provided with two chocolate sandwich bars (125 kcal each) and were given the option to eat both, one, or none of these. Per 100 g, the ready meal provided 160 kcal, 15 g carbohydrate, 6 g protein and 8 g fat, and the chocolate bars provided 435 kcal, 51 g carbohydrate, 5 g protein and 23 g fat. Participants were asked to consume exactly 300 ml of water, but no other food or drink, following the evening meal and before going to bed. On the morning of the study visit, participants were asked to consume exactly 200 ml of water, but no other food or drink, before arrival. Participants refrained from strenuous exercise and alcohol consumption the day prior to the study visit, as both are evidenced to modify appetite regulation [18]. Participants were asked to arrive for the study visit in plenty of time to start at 07.15 (group 1) or 07.45 (groups 2–4). A standardised breakfast was then provided at precisely 07.45 or 08.15, respectively. Participants remained in the clinical unit for the entirety of the study visit, to limit external influencing factors, including physical activity and environmental conditions. A schematic representation of study protocols 1 and 2 are shown in Fig. 3. Two researchers were present at each study visit and a maximum of two participants were in attendance each day.

### Preload and study meals

As shown in Fig. 2, all male participants (group 1 and group 2) and 10 female participants (group 3) received a 266 kcal toast, butter and jam breakfast meal, equivalent to ~ 10% of the recommended male daily energy intake and ~ 12% of the recommended female daily energy intake [11]. Female participants in group 4 received a lower energy 214 kcal toast, butter and jam breakfast, equivalent to ~ 10% of the recommended female daily energy intake [11]. Per 100 g, the breakfast meal provided 241 kcal, 40 g carbohydrate, 7 g protein and 5 g fat. A breakfast of toast and jam was chosen for this pilot study as it is a popular breakfast choice, was likely to be well tolerated by participants, and was a quantity that, according to the researchers, could ensure that participants would be suitably hungry at the ad libitum lunch meal. Two hours after receiving the breakfast meal, all participants consumed a 250-ml preload drink of commercially available orange juice (117 kcal). A pasta-based lunch meal was served ad libitum to the participants 2 h (group 1) or 2.5 h (groups 2, 3 and 4) after the preload*.* Per 100 g, the lunch meal provided 141 kcal, 22 g carbohydrate, 5 g protein and 3 g fat. The pasta meal was cooked in batches of dry farfalle pasta (500 g), microwavable tomato and basil sauce (760 g), olive oil (40 ml) and grated parmesan cheese (50 g). Participants consumed the lunch meal in a laboratory controlled isolated eating environment. Whilst this is not a normal eating environment, eating in the company of other people is known to influence eating behaviours, which would increase the variability in food intake [19]. Before commencing the meal, the researcher instructed the participant to “continue eating until you feel comfortably satisfied”. Pasta (approximately 400–500 g) was served warm in weighed bowls. Every 5 min a fresh bowl of pasta was provided to the participant and the bowl containing the leftover pasta was removed. Bowls were changed every 5 min so that participants were not presented with a ‘set portion’ of food [20]. Bowls containing the leftover pasta were re-weighed. The total amount of pasta and energy consumed at the lunch meal were then calculated. During the visit, water was provided to the participants in 200 ml servings at the times shown in Fig. 3.

### Appetite and palatability measurements

At pre-determined intervals during the study day (as shown in Fig. 3), participants completed paper-based questionnaires that included a series of 100 mm visual analogue scale (VAS) on satiety and motivation-to-eat, which were based on those validated by Flint et al. [21]. The questionnaire assessed hunger, desire-to-eat, satiety, fullness and prospective food consumption (PFC) through the following questions: how hungry do you feel?; how strong is your desire to eat?; how satiated (i.e. pleasantly satisfied) are you?; how full do you feel?; how much food do you think you can (or would want to) eat? Questions were anchored at 0 mm with ‘not at all’ and at 100 mm with ‘extremely’. Following each study meal (breakfast, preload and lunch), participants also completed a VAS questionnaire on the palatability of the study meal/preload provided. This included questions on perceived taste, palatability and enjoyment. For each VAS question, a numerical score of between 0 and 100 mm was obtained.

### Statistical analysis

A sample size of 10 participants per group was chosen based on guidelines for pilot studies by Isaac and Michael [22]; however, a formal a priori power calculation was not performed. All data were analysed using Predictive Analytics Software version 21.0 for Windows (SPSS Inc., Somers, NY, USA). Data were checked for normal distribution using the Shapiro-Wilk test. Appetite score (mm) was calculated as [desire to eat + hunger + (100 − fullness) + prospective food consumption]/4 [23–25]. Area under the curve (AUC) values were calculated for satiety and motivation-to-eat ratings for the following segments using the trapezoid method: breakfast to preload (90 min); post preload (45 min); before lunch (15 min); post lunch (15 min) and total (165 min). Segmental AUC values were analysed by a one-way ANOVA, with pre-breakfast values, BMI category (normal weight or overweight) and stage of menstrual cycle (females only) used as covariates. Pearson’s correlation analysis was used to determine (i) the correlation between the different VAS measures of satiety and motivation-to-eat, and the correlation in appetite score ratings between the groups. Group differences in ad libitum energy intake at lunch, and palatability of study food and drinks, were analysed by one-way ANOVA. Bonferroni correction was used for multiple comparisons when significant differences were detected. Statistical significance was accepted at the 5% level. Data presented as means ± standard deviation (SD).

## Results

Participants anthropometric, blood pressure and three-factor eating questionnaire (TFEQ) data is provided in Table 1.

**Table 1:** Participant baseline data

	Group 1 (male participants, 266 kcal breakfast, protocol 1)	Group 2 (male participants, 266 kcal breakfast, protocol 2)	Group 3 (female participants, 266 kcal breakfast, protocol 2)	Group 4 (female participants, 214 kcal breakfast, protocol 2)
Gender				
Male (*n*)	10	10	0	0
Female (*n*)	0	0	10	10
Age (years)	30 ± 5	26 ± 3	26 ± 7	29 ± 7
Bodyweight (kg)	79.2 ± 8.3	74.2 ± 6.6	67.7 ± 5.2	66.5 ± 11.6
BMI (kg/m^2^)	24.1 ± 1.5	23.1 ± 1.5	23.6 ± 2.0	23.7 ± 2.3
Waist circumference (cm)	86 ± 6	84 ± 5	79 ± 6	81 ± 9
Body fat (%)	17.0 ± 4.4	14.4 ± 3.0	30.9 ± 4.2	30.9 ± 5.0
Systolic blood pressure (mmHg)	120 ± 5	128 ± 6	117 ± 9	115 ± 12
Diastolic blood pressure (mmHg)	71 ± 5	74 ± 7	72 ± 6	71 ± 7
TFEQ				
Cognitive restraint (score out of 21)	6 ± 3	5 ± 4	8 ± 5	10 ± 4
Disinhibition/emotional eating (score out of 16)	5 ± 2	5 ± 3	7 ± 3	6 ± 2
Hunger, measure of feeling hungry (score out of 14)	6 ± 1	6 ± 2	8 ± 3	6 ± 4

Participant baseline data. Data separated into the four study groups and presented as means ± standard deviation. *TFEQ* three-factor eating questionnaire

### Appetite responses

Correlations between specific VAS measures of satiety and motivation-to-eat are shown in Table 2. Significant, positive correlations were found between hunger, desire-to-eat and PFC, and between satiety and fullness measures. Significant, negative correlations were found between satiety (satiety and fullness) and motivation-to-eat (hunger, desire-to-eat and PFC) measures.

**Table 2:** Pearson correlations between the different satiety and motivation-to-eat measures

	Hunger	Satiety	Fullness	Desire-to-eat	PFC
Hunger	1.000	− 0.781*	− 0.787*	0.821*	0.846*
Satiety	− 0.781*	1.000	0.810*	− 0.633*	− 0.710*
Fullness	− 0.787*	0.810*	1.000	− 0.687*	− 0.780*
Desire-to-eat	0.821*	− 0.633*	− 0.687*	1.000	0.752*
PFC	0.846*	− 0.710*	− 0.780*	0.752*	1.000

*Significant positive or negative correlation (*P* < 0.001)

**Fig. 4:**
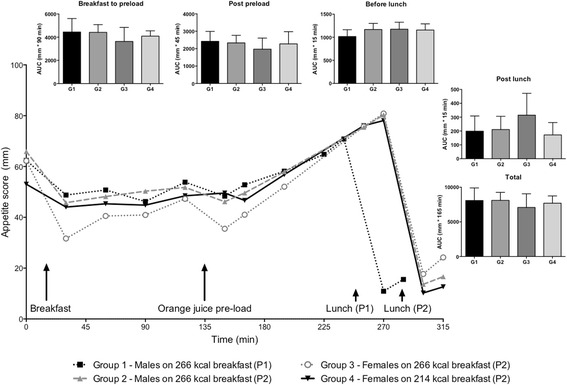
Appetite score data shown as individual time points and segmental AUC. G1 = group 1; G2 = group 2; G3 = group 3; G4 = group 4. P1 = protocol 1 and P2 = protocol 2. Data provided as means ± standard deviation

AUC measures of hunger, satiety, fullness, desire-to-eat and prospective food consumption were not affected by energy intake at breakfast or the two different protocols. As shown in Fig. 4, there was a trend for lower total AUC appetite scores in group 3 (female participants on the 266-kcal breakfast) compared to the other study groups, and for AUC breakfast to preload and AUC post preload, however these did not reach statistical significance. This is also reflected in line graph of Fig. 4, which clearly shows a trend for a lower appetite score at individual time points, in group 3, compared to the other groups, from breakfast to post preload. Conversely, there was a trend for higher AUC appetite score post lunch in group 3, however, again, this was not statistically significant.

Appetite score ratings for male participants following protocol 2 (group 2) were more strongly correlated with the appetite score ratings of female participants following protocol 2 given a gender-specific breakfast (group 4) (*r* = 0.747) (Pearson’s correlation analysis), compared to a non-gender-specific breakfast (group 3) (*r* = 0.479).

### Energy intake at lunch

**Fig. 5:**
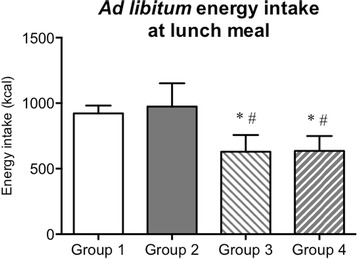
Ad libitum energy intake at the pasta-based lunch meal. Group 1, 10 males following protocol 1 and 266 kcal breakfast; group 2, 10 males following protocol 2 and 266 kcal breakfast; group 3, 10 females following protocol 2 and 266 kcal breakfast; group 4, 10 females following protocol 2 and 214 kcal breakfast. *Significantly different from group 1 (*P* = 0.003), ^#^significantly different from group 2 (*P* = 0.001). Data presented as mean ± standard deviation

As shown in Fig. 5, no significant differences in ad libitum energy intake at lunch were found between group 1 (males following protocol 1) and group 2 (males following protoocl 2). Moreover, no differences were found between groups 3 and 4 for energy intake at lunch, revealing that energy intake at breakfast did not affect energy intake at lunch in the female participants. Males consumed more energy at lunch than females, with significant differences between groups 1 and 3 (*P* = 0.003), groups 1 and 4 (*P* = 0.005), groups 2 and 3 (*P* = 0.002) and between groups 2 and 4 (*P* = 0.003).

### Study meal and preload palatability

No significant differences were found between groups for any measure of food palatability or appearance (scored out of 100 mm) at breakfast, preload or lunch meals. Mean palatability of the study meals was found to be 67 ± 20, 70 ± 18 and 71 ± 17 for the breakfast, preload and lunch meals respectively. Participants’ enjoyment of the study meals was calculated as 64 ± 20, 66 ± 18 and 72 ± 18 for the breakfast, preload and lunch meals, respectively. Participants rated the taste of the study meals as 71 ± 16, 67 ± 21 and 71 ± 16 for the breakfast, preload and lunch meals, respectively.

## Discussion

The aim of this pilot study was to optimise methods for a future preload investigation designed to explore the satiating effects of a non-digestible carbohydrate. Whilst no data was collected specifically to test study acceptability, we believe that fundamental aspects of the study protocol, including questionnaires, clinical environment, and study logistics, were well accepted by both the researchers and the participants. Moreover, all study meals were well received, with mean scores of food/drink enjoyment, palatability and liking, all > 66 out of 100. These meals are therefore suitable for future studies. The palatability and liking of ad libitum meals is especially important to ensure that true satiation is reached rather than a cessation of the meal due to a disliking of the foods provided [7].

The study investigated the satiety effects of altering energy intake at breakfast (gender-specific compared to non-gender specific) and study timings on subjective satiety and ad libitum energy intake at the ad libitum lunch meal. Neither energy intake at breakfast nor the different study timings, significantly altered any of the subjective measures of appetite (hunger, satiety, desire-to-eat, prospective food consumption, fullness or appetite score) or ad libitum intake at lunch.

As previously reported, there were strong correlations between the VAS measures of appetite used in this study [26, 27]. Interestingly, appetite score ratings were more closely correlated between male and female participants when female participants consumed a gender-specific energy intake at breakfast of 214 kcal compared to a non-gender-specific energy intake at breakfast of 266 kcal. Thus, it might be desirable in future mixed-gender studies to adjust the energy provision at the study meals, notably the breakfast meal, to reflect differences in recommended dietary intakes of men and women. An alternative option, and something that was not investigated in the current study, is to provide an energy intake and type of breakfast food that reflects individual differences in habitual intake. Indeed, some studies have accounted for differences in habitual breakfast energy intake, and thus potential gender-specific variations, by asking the participants to self-select their own breakfast portion at the first study visit and then standardise this across all other study visits [28, 29].

Provision of a standardised meal the day before each study visit provides greater control of nutritional intakes of the participants prior to each visit; however, it does mean that intakes may not be reflective of habitual intake. Here, energy content of the evening study meal was not adjusted for gender differences. In future investigations, the provision of a gender-specific energy intake at the evening meal may further improve the correlation in appetite measures between males and females during the study visit [28]. In the current study, participants were given the option of 2, 1 or none chocolate bars with the ready meal. This choice should be removed in future studies to standardise this meal across study visits.

The authors are aware of several limitations of this pilot study. Firstly, the low number of participants (10 in each arm) and the parallel study design, meant that the study lacked statistical power. Importantly, the number of participants required to achieve the same statistical power is considerably less with a within subject (paired) study design [21]. However, cross-over design studies have their own potential drawbacks in satiety research. Most notably, in an ad libitum meal scenario, it has been shown that participants consume more food at a second study visit when compared to the first study visit [30]. This carry-over effect is likely due to familiarity with the food/eating environment. Another limitation of the current study, is that while it investigated the effects of the different protocols (1 and 2) on appetite responses, it is probable that the preload to lunch durations investigated here were too similar (only 30 min different) to observe any significant differences in subjective measures or ad libitum energy intake. Nevertheless, the main objectives of this pilot study were to test logistics and assemble data for optimisation of future studies.

## Conclusions

The current pilot study was an important step in the development of a future satiety preload intervention study. In conclusion, the methods utilised in this pilot study are acceptable for future use. Moreover, a gender-specific energy intake at breakfast improved correlation in appetite scores between male and female participants, which is especially important if mixed populations are being investigated. Accordingly, a gender-specific, or individual-specific, energy intake at breakfast will be utilised in future investigations. Preload to ad libitum meal duration, and time of ad libitum meal provision, were not found to alter satiety or ad libitum energy intake; however, this is not surprising given the close similarities in protocols used and a lack of statistical power.

## Abbreviations

ANOVA: Analysis of variance
AUC: Area under the curve
BMI: Body mass index
PFC: Prospective food consumption
SD: Standard deviation
TFEQ: Three-factor eating questionnaire
VAS: Visual analogue scales
